# Inhibition of the fibroblast growth factor receptor (FGFR) pathway: the current landscape and barriers to clinical application

**DOI:** 10.18632/oncotarget.14109

**Published:** 2016-12-22

**Authors:** Young Kwang Chae, Keerthi Ranganath, Peter S. Hammerman, Christos Vaklavas, Nisha Mohindra, Aparna Kalyan, Maria Matsangou, Ricardo Costa, Benedito Carneiro, Victoria M. Villaflor, Massimo Cristofanilli, Francis J. Giles

**Affiliations:** ^1^ Developmental Therapeutics Program of the Division of Hematology Oncology, Chicago, IL, USA; ^2^ Robert H. Lurie Comprehensive Cancer Center of Northwestern University, Chicago, IL, USA; ^3^ Northwestern University Feinberg School of Medicine, Chicago, IL, USA; ^4^ Dana Farber Cancer Institute, Boston, MA, USA; ^5^ Division of Hematology Oncology, University of Alabama Birmingham, Birmingham, AL, USA

**Keywords:** fibroblast growth factor receptor inhibition

## Abstract

The fibroblast growth factor/fibroblast growth factor receptor (FGF/FGFR) is a tyrosine kinase signaling pathway that has a fundamental role in many biologic processes including embryonic development, tissue regeneration, and angiogenesis. Increasing evidence indicates that this pathway plays a critical role in oncogenesis via gene amplification, activating mutations, or translocation in tumors of various histologies. With multiplex sequencing technology, the detection of FGFR aberrations has become more common and is tied to cancer cell proliferation, resistance to anticancer therapies, and neoangiogenesis. Inhibition of FGFR signaling appears promising in preclinical studies, suggesting a pathway of clinical interest in the development of targeted therapy. Phase I trials have demonstrated a manageable toxicity profile. Currently, there are multiple FGFR inhibitors under study with many non-selective (multi-kinase) inhibitors demonstrating limited clinical responses. As we progress from the first generation of non-selective drugs to the second generation of selective FGFR inhibitors, it is clear that FGFR aberrations do not behave uniformly across cancer types; thus, a deeper understanding of biomarker strategies is undoubtedly warranted. This review aims to consolidate data from recent clinical trials with a focus on selective FGFR inhibitors. As Phase II clinical trials emerge, concentration on patient selection as it pertains to predicting response to therapy, feasible methods for overcoming toxicity, and the likelihood of combination therapies should be utilized. We will also discuss qualities that may be desirable in future generations of FGFR inhibitors, with the hope that overcoming these current barriers will expedite the availability of this novel class of medications.

## INTRODUCTION

The fibroblast growth factor/fibroblast growth factor receptor (FGF/FGFR) is a receptor tyrosine kinase (RTK) signaling pathway that fundamentally regulates embryogenesis, angiogenesis, tissue homeostasis, and wound repair [1, 2]. It also plays important roles in diverse cell functions, including proliferation, differentiation, apoptosis and migration [3-5]. Many prior studies indicate that alterations in FGFR signaling are associated with a broad range of congenital craniofacial developmental disorders. Relatively recently, we have come to understand that somatic mutations of FGFR also have a role in oncologic evolution which makes this pathway of interest when exploring the realm of cancer-directed therapy. Tyrosine kinase signaling pathways have been successfully targeted in malignancies, examples include EGFR in non-small cell lung cancer (NSCLC) [6], KIT in gastrointestinal stromal tumors [7], and ERBB2 breast cancers [8], to name a few.

In this review we will assess the current state of clinical trials involving FGFR directed therapy, discuss the limitations of selecting appropriate patient populations, explore likely side effects to these medications along with methods to counteract them, and propose qualities necessary for subsequent generations of FGFR/FGF pathway drugs. We ultimately need to re-evaluate how we can better develop strategies to bring direct FGFR inhibitors into the clinical setting.

## FGF/FGFR SIGNALING PATHWAY

The FGF family contains 22 known ligands and FGFs interact with the extracellular matrix as well as the cell surface *via* stabilization by heparan sulphate proteoglycans (HSPGs). The communications of FGFs with HSPGs has been shown to be essential for FGF signal transduction [9]. In comparison, there are only 4 highly conserved transmembrane tyrosine kinase receptors (FGFR1-4) identified in the FGFR family. The members differ from one another in their ligand affinities and tissue distribution with variations in splicing of FGFR1-3 accounting for some additional diversity [10-13]. The fifth related receptor, FGFR5 (also known as FGFRL1), can bind FGFs but has no tyrosine kinase domain and its role in cellular transduction remains unclear [14, 15]. Though there is no concrete evidence, it is hypothesized that FGFRL1 may serve as a ligand trap and bind FGFs, may dimerize with other transmembrane FGFRs and inhibit autophosphorylation, or may increase turnover rates of other FGFRs [16].

**Figure 1:**
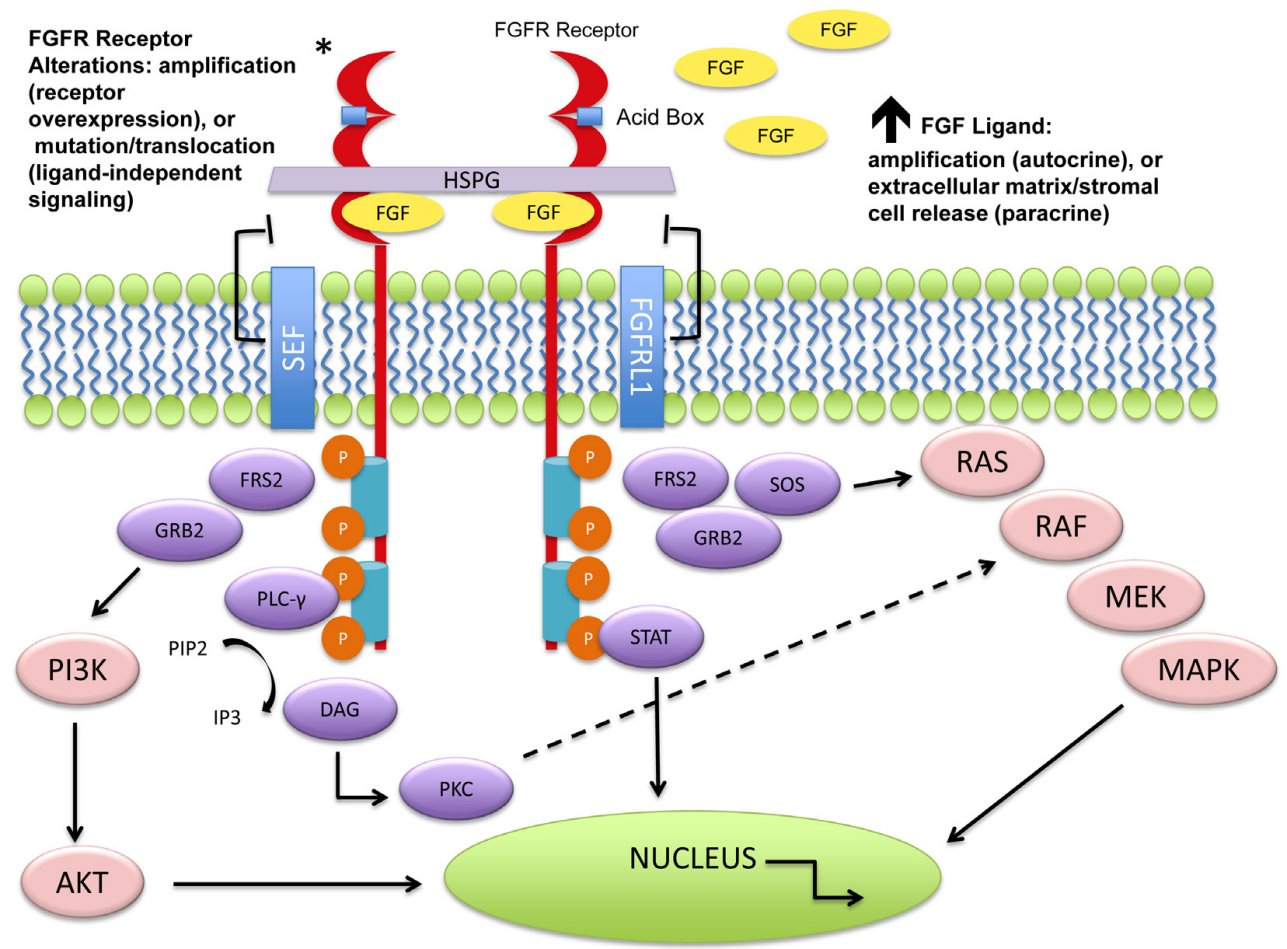
Molecular aberrations leading to FGFR pathway activation The FGFRs dimerize upon ligand binding and trigger a downstream cascade of signaling pathways. The FGFR receptors (1-4) can become activated by mutation, translocation, or gene amplification. An increase in circulating FGF ligands can also cause activation. Downstream signaling can trigger the mitogen activated protein kinase (MAPK) pathway, the phosphoinositide-3-kinase (PI3K/Akt) pathway, the phosphorylation of the signal transducer and activator of transcription (STAT), and the PLCγ activation of the DAG-PKC and IP3-Ca^2+^ cascade resulting in DNA transcription. Negative feedback loops can attenuate the signaling cascade at varying levels. As seen above, the “similar expression to FGF” (SEF) family members can interact with the cytoplasmic domain of FGFRs and inhibit downstream signaling. It is hypothesized that FGFRL1 (atypical receptor/FGFR5) may serve as a ligand trap, may dimerize with other transmembrane FGFRs and inhibit autophosphorylation, or may increase turnover rates of other FGFRs [16]. No evidence exists for these mechanisms.

Upon ligand binding, FGFRs dimerize and trigger a cascade of downstream signaling pathways, including the mitogen activated protein kinase (MAPK), signal transducer and activator of transcription (STAT), the phosphoinositide-3-kinase (PI3K)/Akt pathways, and DAG-PKC and IP3-Ca^2+^ signaling branches *via* PLCγ activation [17-20]. The FGFR signaling pathway represents a major target for cancer therapeutics as a number of studies indicate that it plays a crucial role in tumor proliferation, angiogenesis, migration, and survival.

## DEREGULATION OF FGFR SIGNALING IN CANCER

There are several proposed mechanisms for FGFR related oncogenesis including: (i) activating or “driver” mutations resulting in cell growth and survival; (ii) neo-angiogenesis; and (iii) acquired resistance to other cancer therapy [21].

The FGFR pathway is subject to various somatic aberrations resulting in carcinogenesis. Receptor overexpression can be a result of gene amplification or changes in post-transcriptional processing; point mutations may result in constitutive receptor activation or decreased sensitivity to ligand binding; translocations can produce fusion proteins with constitutive activity; and isoform switching and alternative splicing can reduce specificity to FGFs [22]. These major oncogenic aberrations represent features that make FGFR an ideal therapeutic target for treating a broad scope of malignancies.

## FGFR AMPLIFICATION

Using next generation sequencing (NGS) to detect FGFR anomalies, a comprehensive review of a cohort of nearly 5,000 cancer patients found aberrations in 7.1% of malignancies. FGFR1 amplification was the most common abnormality within the overall scope of FGFR anomalies; notably FGFR4 was also seen to have high rates of amplification [23].

### FGFR1

Amplification of the chromosomal region 8p11-12, the genomic location of FGFR1, has been detected in 10% of breast cancers (predominantly in estrogen receptor (ER) positive cancers) and this finding has been related to higher FGFR1 expression levels correlating to worse prognosis [24]. Recently, it has also been reported that FGFR1 is amplified in as many as 19% of squamous non-small cell lung cancers (SqCLC) [25]. Moreover, preclinical studies have shown that a subset of FGFR1-amplified small cell lung cancer is extremely sensitive to FGFR inhibition by PD173074, a specific FGFR1 inhibitor [26]. FGFR1 amplifications have also been reported in oral squamous cell carcinoma [27], ovarian cancer [28], bladder cancer [29] and rhabdomyosarcoma [30].

### FGFR3

In a study using microarray-based comparative genomic hybridization on samples from 18 patients with recurrent or metastatic adenoid cystic carcinoma (ACC), the investigators noted that 61% of tumors displayed a DNA copy number increase that was associated with loci for FGF(R)s. In particular, a gain at the region for FGFR3 was detected more frequently in clinically aggressive tumors [31]. Interestingly, earlier work in ACC had identified FGFR1 overexpression by immunohistochemistry, which was thought to have a role in neoplastic progression as well though this was not seen with the microarray analysis [32]. Interestingly, a phase II clinical trial in 32 patients with ACC demonstrating clinical progression on standard therapy used the pan-FGFR inhibitor dovitinib (NCT01417143) and demonstrated modest anti-tumor activity [33].

### FGFR 4

Amplification and activating mutations in FGFR4 have been identified in 7-8% of rhabdomyosarcoma patients and FGFR inhibitors are potentially effective in a rhabdomyosarcoma mouse model expressing mutated FGFR4 [34]. A variety of preclinical studies have shown FGFR4 overexpression with a role in prostate cancers [35], colon cancers [36], and liver cancers [37]. FGFR4 is the only FGFR receptor expressed in mature hepatocytes and in a subset of patients with HCC (~30%), overexpression of both FGF19 and FGFR4 is observed. Among the physiologic functions of the FGF19-FGFR4 axis is a major role in the regulation of hepatic bile acids *via* a negative feedback system controlling *de novo* hepatic bile acid synthesis. In patients with extrahepatic cholestasis, plasma levels of FGF19 are elevated which is consistent with our understanding of this pathway. Inhibition of this axis is thus hypothesized to result in a disruption of bile acid homeostasis [38].

**Figure 2:**
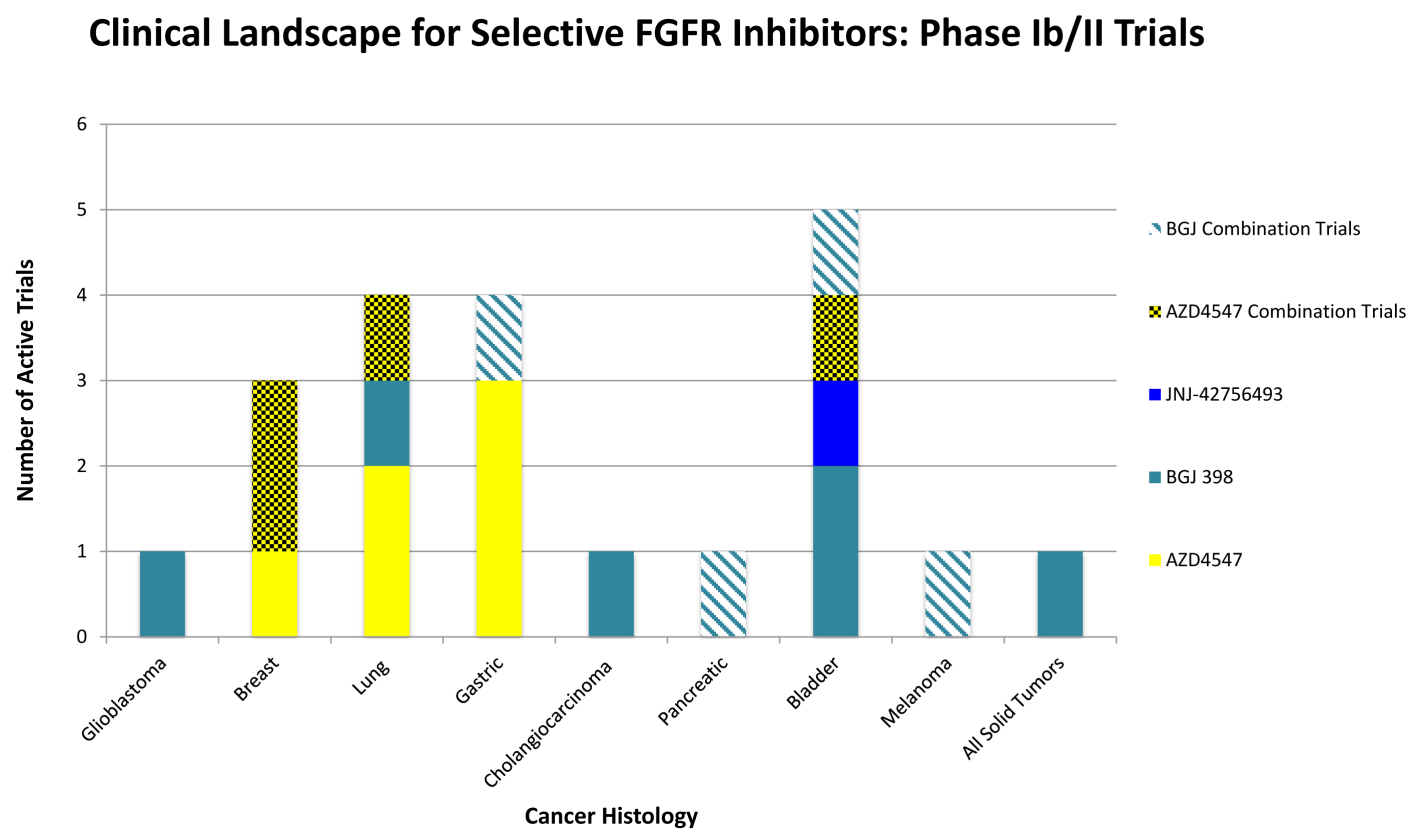
Selected overview of phase II clinical trials evaluating FGFR Inhibitors as monotherapy or in combination with existing therapies Bladder, Lung, and Gastric cancers all are areas of interest in advanced clinical trials testing FGFR pathway inhibition. There exist a wide scope of histologies where FGFR inhibition may be of clinical benefit, many phase II trials are currently in the recruiting stages or are actively ongoing.

## FGFR MUTATION

The same review mentioned above also noted that gene mutations and rearrangements affecting FGF/FGFR signaling were less common than amplification [23].

### FGFR 2

Mutations in FGFR 2 are implicated in a broad spectrum of malignant disease. Mutations are present in 12% of endometrial carcinomas and FGFR2 mutant endometrial cancer cell lines are highly sensitive to FGFR tyrosine kinase inhibitors, implicating FGFR2 as an innovative therapeutic target in endometrial carcinoma [39]. Also, approximately 10% of cases of gastric cancer are associated with FGFR2 amplification and/or mutation; in particular amplification is suggestive of a poor prognosis and more widespread disease [40]. Gastric cancer cell lines with FGFR2 amplifications show evidence of ligand-independent signaling and are highly sensitive to FGFR inhibitors [41]. In breast cancer, single nucleotide polymorphisms (SNPs) in FGFR2 were found to be strongly associated with evidence of postmenopausal disease [42]. FGFR2 amplification is also detected in 5% of triple-negative breast cancers, providing the possibility of specific targeted therapy when many other options are less efficacious by the nature of the disease profile [43]. Recently, several novel FGFR2 mutations have been identified in lung cancer, both in cases of adenocarcinoma and squamous cell carcinoma [44, 45]. *In vitro* and *in vivo* (xenograft mouse model) evaluation has demonstrated oncogenic potential, with increased cell growth thought to be a result of ligand-independent dimerization leading to constitutive receptor activation [45]. Use of pan-FGFR kinase inhibitors was noted to result in inhibition of cell growth [44, 45].

As aforementioned, FGFR mutations do not always result in “driver” mutations alone. As seen 10% of melanoma, missense mutations of FGFR2 have been identified in BRAF-inhibitor treatment resistance [46, 47].

### FGFR3

FGFR3 mutations are found in approximately 70% of non-muscle-invasive bladder cancers and 10-20% of invasive bladder cancers [48]. The presence of an FGFR3 mutation strongly relates to low-grade, non-muscle-invasive tumors with a better prognosis, however the clinical viability of FGFR3 as a target for cancer directed therapy in this population is unclear and remains controversial [48]. Interestingly, in patients with non-invasive bladder cancer after resection, the presence of an FGFR3 mutation in cells obtained from urinalysis at routine follow-up was predictive of disease recurrence [49]. FGFR3 mutations have also been identified in many other cancer types, including 3% of squamous cell lung carcinoma [23], cervical cancers [50], multiple myeloma [51], prostate cancer [52] and spermatocytic seminomas [53]. In head and neck squamous cell carcinoma (HNSCC) that was positive for human papilloma virus (HPV, 42.5% of 120 tumor samples), genomic analysis using parallel sequencing technology revealed nearly 18% of tumors with mutations in FGFR2 or FGFR 3, which was notably different than in HPV negative samples [54]. Recent investigation attempted to further elucidate the relevance and prognostic significance of FGFR3 mutation in HNSCC. Interestingly, HPV negative cases that had FGFR3 mutations were not as responsive to FGFR inhibition as the single HPV positive case studied, suggesting further need for study in HNSCC based on HPV status [55]. FGFR3-activating mutations are also found at a high frequency in epidermal nevi and seborrhoeic keratosis, which are benign skin conditions and do not progress to malignancy [56, 57]. In contrast to the activation of FGFR3 by mutation, amplifications of FGFR3 have been rarely described in cancers.

## FGFR REARRANGEMENTS/FUSION

Of the 4 FGFR receptors, FGFR2 and FGFR3 are identified as having comparatively more frequent gene rearrangements [23]. In a comprehensive survey of gene fusions across different solid tumor histologies, the authors described a wide-ranging distribution of FGFR1, FGFR2, and FGFR3 fusions across 8 of 20 tumor types analyzed [58].

### FGFR 1

The 8p11 myeloproliferative syndrome (EMS) demonstrates peripheral blood leukocytosis with eosinophilia, myeloid hyperplasia of the bone marrow, and T-cell lymphoblastic leukemia/lymphoma. Clinically, EMS is overall aggressive with a brief chronic phase prior to rapid transformation into acute leukemia. All cases show a chromosomal abnormality implicating the FGFR1 gene at chromosome 8p11. The novel chimeric activated fusion proteins consist of an N-terminal portion with a dimerization domain and the C-terminal portion that houses the FGFR1 tyrosine kinase domain [59]. Pre-clinical evidence suggests that FGFR inhibitors are able to reduce growth and induce apoptosis in cell lines harboring FGFR1 gene rearrangements [60].

### FGFR 2

Intrahepatic cholangiocarcinoma demonstrates FGFR2 fusions in 13.6% of cases that are mutually exclusive with KRAS/BRAF mutations, and *in vivo* cellular studies confirm the oncogenic potential of this aberration. These studies also indicated a potent sensitivity to FGFR inhibitors [61].

**Table 1:** Main FGFR Genomic Alterations Found in Human Cancers

Gene	Molecular Alteration	Cancer type (prevalence; reference)
**FGFR1**	**Amplification**	Squamous NSCLC (20%; [25]) Breast cancer (10%; [24]) Ovarian cancer (~5%; [28]) Bladder cancer (3%; [29]) Others: oral squamous cell carcinoma, esophageal squamous carcinoma ([22, 27])
	**Mutation**	Melanoma (rare) Glioblastoma ([63]) Pilocytic astrocytoma (5-8%, [21])
	**Translocation**	8p11 myeloproliferative syndrome Chronic myeloid leukemia (rare; [59, 60])
**FGFR2**	**Amplification**	Gastric cancer (5-10%; [40, 86]) Breast cancer (4% of triple negative cases; [42, 43])
	**Mutation**	Endometrial cancer (12%; [39]) SqNSCLC (3%; [23]) Melanoma (may be loss of function, [104])
	**Germline SNP**	Second intron SNP; breast cancer susceptibility ([42])
**FGFR3**	**Amplification**	Bladder cancer ([10]) Salivary adenoid cystic cancer ([10])
	**Mutation**	Bladder cancer (50%-60% non-muscle invasive; 10%-15% muscle invasive; [21]) Cervical cancer (5%; [10]) Myeloma (5% of the translocated cases; [51]) Spermatocytic seminoma (7%; [53])
	**Translocation**	Myeloma (15%-20%; [10, 21])
**FGFR4**	**Amplification/Mutation**	Rhabdomyosarcoma (7%-8%; [21, 34])
	**Germline SNP**	Coding SNP; poor prognosis in many cancer types [10]

### FGFR 3

It is has been shown that nearly 15% to 20% of multiple myeloma cases involve the chromosomal translocation t(4;14), bringing FGFR3 and the adjacent multiple myeloma SET domain (MMSET) gene under the control of the Ig heavy chain promoter. This leads to the aberrant expression of FGFR3 and MMSET [51, 62]. It is important to note that chromosomal translocation t(4;14) myeloma cell lines are highly sensitive to FGFR3 targeting inhibition [62]. Fusion aberrations have been described in FGFR 1-3 genes with multiple partners (examples include TACC1, TACC2, TACC3, BAIAP2L1, NPM1, AFF3) across a wide spectrum of tumor histologies. The TACC3 gene (transforming acidic coiled-coil containing protein) was first identified as a component of FGFR3-TACC3 fusion in glioblastoma multiforme (GBM) and bladder urothelial tumors, this fusion protein is constitutively active and has been shown to affect mitosis by altering chromosomal segregation patterns [63, 64]. In an analysis of nearly 600 cases of lung adenocarcinoma patients without any smoking history, investigators found an FGFR3-TACC3 fusion in a tissue sample from a patient that previously did not have any known oncogenic alteration. As a whole, tumors harboring FGFR3-TACC3 were identified in 0.5% of the entire cohort (including those cases with known oncogenic mutations such as EGFR). *In vitro*, these cells with an FGFR3-TACC3 fusion demonstrated sensitivity to pan-FGFR inhibitors, suggesting a possible subset of lung adenocarcinoma patients that may benefit from targeting this pathway [65]. It is also worth mentioning that we described the first 3 cases of cervical cancer harboring the FGFR-TACC3 fusion, noting that one patient received treatment with FGFR targeted therapy and achieved stable disease for 4 cycles [64, 66].

## AUTOCRINE/PARACRINE SIGNALING

Most of the genetic aberrations discussed above lead to constitutive receptor activation and ligand-independent signaling. However, ligand-dependent signaling may also occur and would suggest that ectopic expression of FGFs can promote cancer. Many preclinical models have done exactly this with the expression of FGF in either cancer cells or stromal cells, showing the autocrine and paracrine stimulation of cancer cells, respectively. Evidence of these autocrine and paracrine circuits have been identified in melanomas [67], in NSCLC [68], prostate adenocarcinomas [69], and in triple negative breast cancers [70]. Increased FGF release from stromal or tumor cells may have a role in cell survival, proliferation, and angiogenesis.

## ANTI-FGF/FGFR THERAPEUTIC APPROACHES

In an effort to capitalize on FGF/FGFR signaling in tumorigenesis, a number of novel drugs targeting the FGF/FGFR cascades have been introduced and are currently undergoing preclinical and clinical trials in various FGFR-related tumors. Early development of FGFR inhibitors exhibits antitumor activity and present very specific toxicity profiles. Prior studies also indicate that FGFR inhibitors enhance tumor sensitivity to conventional anticancer drugs such as 5-fluorouracil, irinotecan, paclitaxel, and etoposide in human cancer cells acquiring anti-apoptotic potential based on aberrant FGFR activation [71, 72].

Current FGFR inhibitors can be divided into groups according to the mechanism of action: (i) small molecules, which are commonly classified as receptor tyrosine kinase inhibitors (TKIs). TKIs are mainly ATP-competitive molecules binding to the cytoplasmic kinase domain and either inhibit the catalytic activity of FGFRs or the autophosphorylation of tyrosine residues; (ii) antagonistic antibody or peptide inhibitors, which bind to the FGFR extracellular domain and compete with FGFs, thereby blocking FGF-FGFR association and FGFR dimerization; (iii) FGF ligand traps, which can potentially block the activity of multiple FGF ligands and receptors, exerting both anti-angiogenic and anti-proliferative effects.

Within the group of small molecule inhibitors, there exist both nonselective and selective FGFR TKIs. Nonselective FGFR TKIs are compounds that bind to the relatively conserved ATP-binding domain in RTKs and as their name implies, lack kinase selectivity. The nonselective FGFR TKIs target other RTKs such as vascular endothelial growth factor receptors (VEGFRs) and platelet-derived growth factor receptors (PDGFRs), and usually present modest bioactivity against the FGFR family. Most of the RTKIs assessed to date are non-selective FGFR inhibitors. Dual inhibition with VEGFRs/PDGFRs has the obvious potential benefit of simultaneously targeting angiogenesis and tumor cell proliferation. However, many of these TKIs with multiple targets are less potent against the FGFR signaling pathway and give rise to a variety of toxic side effects in clinical and preclinical studies, thereby limiting the ability to deliver drugs at doses required for FGFR inhibition [73, 74]. Pan-FGFR inhibitors such as lenvatinib (E7080), ponatinib (AP24534), regorafenib (BAY 73-4506), dovitinib (TKI258), lucitanib (E3810), cediranib (AZD2171), intedanib (BIBF 1120), brivanib (BMS-540215), and others are currently being studied in clinical trials. These agents fall into the spectrum of multi-kinase TKI's which have FGFR as a part of their portfolio of inhibition.

## NON-SELECTIVE FGFR INHIBITORS

Dovitinib (TKI258, Novartis) is an example of a well-studied second-generation non-selective FGFR inhibitor targeting FGFRs and other RTKs. It inhibits both the kinase activity of FGFR1, FGFR2, and FGFR3 in FGFR-amplified breast cancer and the cellular activity of FGFR3 in t(4;14) multiple myeloma in pre-clinical studies [75, 76]. Dovitinib shows high potency against most FGFRs in addition to targeting c-KIT, CSF-1, VEGFRs and PDGFRs. Antitumor activity in advanced renal cell carcinoma *via* inhibition of FGFR1 has been demonstrated in Phase I and II clinical trials [77, 78]. A Phase III trial comparing dovitinib against sorafenib for metastatic renal cell carcinoma in the third line setting included 570 patients and dovitinib was not shown to be superior to sorafenib with respect to progression free survival (PFS) or overall survival (OS) [79]. Interestingly, despite the high potency against FGFRs, one Phase II trial in advanced urothelial carcinoma using dovitinib to treat FGFR3 mutated *versus* FGFR wild-type cancer failed to show a meaningful overall response rate and the study was terminated after concluding that dovitinib has limited single-agent activity in this population [80]. Additional Phase II trials in urothelial bladder cancer are ongoing (NCT01732107). TKI258 recently underwent a series of clinical trials for its safety and efficacy in patients with breast cancer (NCT00958971), endometrial cancer (NCT01379534), and multiple myeloma (NCT01058434).

Lenvatinib (E7080, Eisai) is another multikinase inhibitor, inhibiting FGFR1-4 as well as VEGFR1-3, RET, KIT and PDGFR-β [81]. *In vivo*, lenvatinib shows more potent anti-tumor activity than it does *in vitro*. In a triple negative human breast adenocarcinoma xenograft model, lenvatinib demonstrated significant growth inhibition of primary mammary fat pad tumors, intra-tumoral angiongenesis, lymphangiogenesis, and development of lung and lymph node metastasis [82]. Lenvatinib inhibits FGFR1 with an IC_50_ of 46 nmol/L, which is highly potent at a clinically relevant concentration [81].

A Phase 1 dose-escalation study of lenvatinib in subjects with advanced solid tumors and an expanded cohort of patients with melanoma enrolled 77 subjects and determined a maximum tolerated dose (MTD) of 10mg by mouth twice daily (BID) [83]. The notable toxicities included hypertension (43%), fatigue (42%), and proteinuria (39%), all of whom have been identified in other VEGF inhibitors. GI side effects included nausea (25%). Twelve patients (15.6%) achieved partial response (PR, *n* = 9) or unconfirmed PR (uPR, *n* = 3), and 19 (24.7%) achieved stable disease (SD) ≥23 weeks. The most encouraging tumor response in this cohort was in melanoma, however a promising response was also seen in medullary thyroid cancer.

The FDA approved lenvatinib in 2015 for use in radio-iodine refractory, well-differentiated thyroid carcinoma. Recent data from a Phase I trial studying lenvatinib in combination with carboplatin/paclitaxel in treatment naïve advanced NSCLC, demonstrated a MTD of 4mg by mouth BID, with manageable side effects and encouraging anti-tumor activity [84]. In a cohort of 28 patients, a 68% response rate was noted with a median PFS of 9 months. Biomarkers that correlated with disease response were stromal cell-derived factor 1alpha, stem cell factor, and granulocyte colony-stimulating factor.

Ongoing Phase II/III clinical trials include a comparison of lenvatinib with sorafenib in hepatocellular carcinoma (NCT01761266), lenvatinib with everolimus in renal cell carcinoma (NCT02454478), and as monotherapy in unresectable biliary cancer (NCT02579616).

A further understanding of the predictive significance of various biomarkers is needed, and screening for FGFR in advanced cancer across tumor histologies may play a role in selecting patients likely to have the best response to therapy. We have evidence that lenvatinib has anti-tumor activity across multiple malignant histologies. This should prompt further study, as is not certain if the observed benefit stems mainly from FGFR inhibition or from suppression of many other pathways altogether.

## SELECTIVE FGFR INHIBITORS

Subsequent pharmaceutical development has led to highly selective and highly bioactive FGFR inhibitors (i.e., selective FGFR TKIs). These include compounds like AZD4547, BGJ398, JNJ42756493, and PD173074.

### AZD4547

AZD4547 is a small-molecule compound that is a selective FGFR (FGFR 1-3) inhibitor, delivered orally in capsule form. The drug demonstrated potent inhibition of proliferation in cell lines with activation of the FGFR pathway and also in tumor xenograft models [85]. Oral administration of AZD4547 has also resulted in prolonged survival of FGFR3-TACC3-transformed glioma xenografts by 28 days compared with mice treated with the vehicle control [63]. Furthermore, inhibition with AZD4547 resulted in a significant dose-dependent tumor growth inhibition and survival of gastric cancer carrying an FGFR2 gene amplification both *in vitro* and *in vivo* [86]. Other pre-clinical studies on xenograft models transplanted with transformed cells derived from FGFR1 amplified NSCLC cancer patients have shown that AZD4547 stops tumor growth and promotes regression [87].

A Phase I, open label, multicenter study to assess the safety, tolerability, pharmacokinetics and preliminary anti-tumor activity of ascending doses of AZD4547 in patients with advanced solid malignancies was completed in March 2015 (NCT00979134). A dose escalation study (80 patients) identified 80mg PO BID as the recommended dose. The increase in serum phosphate concentration observed in this phase I study provides evidence that AZD4547 at this dose leads to pharmacologic target inhibition. Dose limiting toxicities reported were elevated liver enzymes, mucositis, stomatitis, renal failure, and hyperphosphatemia. Expansion cohorts to further assess safety and tolerability required tumors with FGFR 1 amplification as confirmed through FISH (FGFR: Centromeric ratio ≥ 2). In a cohort of 15 patients with FGFR1 amplified SqCLC, the most common adverse events (AEs) were dermatologic and GI related. Grade ≥ 3 adverse events (AEs) occurred in 3 patients (central serous retinopathy (CSR), dehydration, hyponatremia). Treatment related severe AEs occurred in 3 patients as well (CSR, asthenia and dyspnea). There were no deaths due to the drug but 3 discontinuations due to AEs [88]. In a cohort of 13 gastroesophageal cancer patients, the reported AEs included vomiting in 8 patients, decreased appetite and diarrhea in 7 patients, fatigue and nausea in 6 patients, hyperphosphatemia, constipation and dry eyes (4 patients each), stomatitis in 5 patients, and retinal pigment epithelial detachment in 4 patients [89].

Of note, partial response (PR, by RECIST criteria) was observed in tumors with a high burden of FGFR aberration including one SqCLC patient with FGFR1 amplification and another patient with FGFR2 amplified gastroesophageal cancer. 4 patients in each cohort were also noted to have stable disease [88, 89].

AZD4547 is currently under a Phase II clinical trial to assess its activity in patients with FGFR1 or FGFR2 amplified breast, squamous lung, and stomach cancer whose cancers have progressed following previous chemotherapy (NCT01795768). 285 patients with advanced cancer were screened, identifying FGFR1 amplification in 18% (20/111) HER2 negative breast cancer, 9.5% (4/42) NSCLC, and FGFR2 amplification in 7.6% (10/132) gastroesophageal (GC). Confirmed RR was 33% (3/9) in FGFR2 amplified GC, and 12.5% (1/8) FGFR1 amplified BC. Similar AEs were reported. These preliminary results indicate that AZD4547 demonstrated high activity in FGFR2 amplified GC and lower activity in FGFR1 amplified BC. The investigators noted that FGFR2 copy number in cell free plasma DNA was elevated in all PR of GCs; this may provide a screening tool to identify FGFR2 amplified GC with likelihood of treatment response [90].

AZD4547 is also being evaluated both against existing therapies and in conjunction with existing therapies in a variety of Phase II trials. A recently completed Phase II trial aimed to evaluate the safety and efficacy of AZD4547 *versus* paclitaxel in advanced gastric or gastro-oesophageal junction cancer; no results have been reported (NTC01457846). AZD4547 is also undergoing a Phase I/II clinical trial in combination with fulvestrant *versus* fulvestrant alone in ER+ breast cancer patients with FGFR1 amplification (NTC01202591).

### BGJ398

BGJ398 (Novartis) is an additional potent, pan-FGFR inhibitor currently undergoing Phase I/II clinical trials after initially demonstrating antitumor activity in RT112 bladder cancer xenografts models overexpressing wild-type FGFR3 [91]. A dose escalation trial is actively ongoing (NCT01004224) and preliminarily has reported a maximum tolerated dose of 125mg/day with a 21-day on/7-day off schedule for dosing based on safety data. Of the 94 enrolled patients initially, partial responses were seen in 4 FGFR3 mutated bladder cancers, 2 FGFR1 amplified SqCLC, and a reduction in tumor burden was seen in FGFR2 fusion cholangiocarcinoma as well as in FGFR1 amplified breast cancer [92]. Investigators used FISH to screen for FGFR1 amplification, and in the cohort of 17 SqCLC (expansion arm) patients there were 4/17 PRs (2 after data cutoff date) and 3 patients with SD. Major adverse effects were reversible hyperphosphatemia, as well as stomatitis, alopecia, decreased appetite, and fatigue [93]. These study results suggest efficacy, and as such, efforts to optimize predictive biomarkers for FGFR inhibitor sensitivity are ongoing. Recently multiple investigators have suggested that high levels of FGFR1 mRNA are more indicative of response to TKIs in pre-clinical models of SCC and a subset of head and neck squamous cell cancers (specifically to BGJ398 in HNSCC) [94, 95]. Trials that are actively recruiting for study of BGJ398 alone exist for non-muscle invasive urothelial carcinoma (NCT02657486), recurrent glioblastoma (NCT01975701), and advanced cholangiocarcinoma (NCT02150967). Additionally, pre-clinical data suggests that gastrointestinal stromal tumors (GIST) demonstrating resistance to imatinib may be secondary to FGF pathway activation [96]; a Phase I/II clinical trial is actively recruiting to evaluate BGJ398 in combination with imatinib in untreated advanced GIST (NCT02257541).

### JNJ-42756493

JNJ-42756493 (Janssen) is another pan-FGFR inhibitor that is orally bioavailable. Initial data from a Phase I trial to evaluate the safety, pharmacokinetics, and pharmacodynamics in adult patients with advanced or refractory solid tumors or lymphoma recommends a 10mg (7 days-on/7 days-off schedule) as the appropriate tolerable dose with clinical response (NCT01703481). Biomarkers in this study included tumor tissue genomic profiling, skin/tumor biopsies and soluble serum markers. Of the 65 patients enrolled, 23 were evaluated for response to treatment and investigators identified 5/23 patients that responded (4 confirmed, and 1 unconfirmed partial response). Stable disease (SD) was seen in 16/23 patients with glioblastoma, urothelial or endometrial cancer. All patients that responded demonstrated FGFR2 or FGFR3 translocations, and of the responses identified, 3 of the patients with partial responses harbored an FGFR3-TACC3 fusion alteration. Similar to other selective FGFR inhibitors, the most common adverse events included hyperphosphatemia (65%), asthenia (55%), dry mouth (45%), nail toxicity (35%), constipation (34%), decreased appetite (32%), and dysgeusia (31%). Dose-dependent elevations in serum phosphate were seen to represent pharmacodynamic effect of the medication [97]. A Phase II trial in urothelial cancer and a Phase I trial in advanced hepatocellular carcinoma are actively recruiting (NCT02365597, NCT02421185).

## MONOCLONAL ANTIBODIES

In addition to RTKIs, several monoclonal antibodies targeting FGF/FGFR are in preclinical or early phase development; these are directed toward a particular FGFR and interfere with ligand binding or receptor dimerization. The goal is to reduce the potential toxicity of pan-FGFR inhibition given the specificity of antibody-antigen interactions.

An FGFR2-IIIb-specific antibody, GP369, has been shown to inhibit the proliferation of human cancer cell lines and tumor xenografts with amplified or activated FGFR2 signaling [98]. BAY1187982 (Bayer) also falls under the spectrum of exploiting the antibody/antigen relationship as a human anti-FGFR2-Ab that is conjugated to a cytotoxic agent (antibody-drug conjugate). Proof of efficacy has been seen in pre-clinical studies demonstrating successful monotherapy for inhibiting tumor growth in gastric and breast cancer xenograft models that demonstrate FGFR2 overexpression [99]. A phase I dose-escalation trial in patients with advanced stage solid tumors known to express FGFR2 had initially set out to establish a MTD in two cohorts, those with triple negative breast cancer and a second group to encompass other tumors expressing FGFR2 (NCT02368951). Unfortunately, for unknown reasons, the trial was recently terminated.

Antibodies targeting FGFR3 have also been shown to have significant inhibitory effect on cell proliferation in bladder cancer cells [100] and t (4; 14)-positive multiple myeloma [101]. MFGR1877S (Genentech) is a human anti-FGFR3 monoclonal antibody that demonstrated activity in preclinical models of urothelial carcinoma harboring FGFR3 overexpression. Subsequently there have been two Phase I trials completed, one in solid tumors (NCT01363024) and one in t(4; 14)-positive multiple myeloma (NCT01122875). Preliminarily results from the solid tumor trial reported 4/10 with SD in urothelial carcinoma. The DLT was noted to be thrombocytopenia in a single patient. A recommended Phase 2 dose (RP2D) was determined, however further plans for study and development remain unknown at this time [102] .

## FGF-LIGAND TRAPS

The third approach to targeting the FGF/FGFR signaling pathway is to impede ligand binding to the receptor itself by developing FGF-ligand traps. FP-1039 (GSK3052230, GlaxoSmithKline) is a soluble fusion protein that consists of extracellular FGFR1-IIIc fused to the Fc domain of IgG1 and hampers binding of FGF1, FGF2, and FGF4 (AACR 2014 Abstract #5449). A phase II trial in patients with endometrial cancer (NCT01244438) was suspended due to lack of viability given that after screening 70 patients, none qualified for the study. A subsequent Phase II trial is currently recruiting and is looking to evaluate FP-1039 in solid tumors alone, or in combination with docetaxel, or paclitaxel and carboplatin (NCT01868022).

## EXPANDING FGFR DIRECTED THERAPIES: FROM THEORY TO PRACTICE

Given the broad scope of malignancies with FGF/FGFR pathway aberrations, proof of concept has been demonstrated for its role as a driver for oncogenesis, as a downstream key player in angiogenesis, and as a pathway responsible for acquired resistance to other anti-cancer therapies. Pre-clinical and clinical studies have shown that cancers harboring FGF/FGFR pathway aberrations are likely to be sensitive to FGFR inhibitors across various histologies. Ongoing Phase I/II clinical trials have demonstrated reduction in disease burden or stable disease, with evidence of dose dependent increase in serum phosphate, FGF23, and Vitamin D levels indicating likely markers to follow if effectively targeting the FGF/FGFR pathway [103].

Although non-selective FGFR inhibitors are approved for various indications, there are currently no selective FGFR inhibitors that are FDA approved. Ongoing clinical trials highlight the barriers to reaching this goal - how to select the right patient population to better achieve clinical disease response, how to predict and bypass mechanisms of acquired resistance, managing the toxicity profile, and utility in combination with existing anti-cancer therapy.

**Table 2:** Selected anti-FGFR Drugs in Clinical Phases of Development

Drug	Histology	Study Phase	Biomarker	Result/ Response	Toxicity	Clinical Trial ID/ Reference
Nonspecific						
Dovitinib (Novartis)	Urothelial Carcinoma	Phase II		Study Terminated	Thrombocytopenia (9%), fatigue (9%), and asthenia (9%)	NCT00790426 [78]
	Urothelial carcinoma	Phase II – Ongoing, Not Recruiting	FGFR3 by IHC and mutation status	8% CR (6-month TURBT confirmed, 0% in IHC+, Mut – and 33% in IHC+, Mut +)	Hypertension (15%), hypertriglyceridemia (15%), hepatotoxicity (1%), stomatitis, rash	NCT01732107 [142]
	GC	Phase II-Recruiting	FGFR2 copy number > 3 by rtPCR			NCT01719549
Specific						
AZD4547 (AstraZeneca)	Advanced Solid Tumors	Phase I Complete	FGFR1 amplification by FISH (FGFR1:CEP8≥ 2)			NCT00979134
	SqCLC Expansion Cohort	Ib-Complete		SqCLC: PR in 1/14, SD 4/14	Central serous retinopathy, dehydration, hyponatremia	[88]
	GC and GEJC	Ib - Complete		GC/GEJC: PR in 1/13, SD in 4/13	Vomiting (61.5%), decreased appetite and diarrhea (53.9%), fatigue and nausea (46.2%), hyperphosphatemia, constipation and dry eye (30.7%), epithelial and mucosal dryness (61.5%), stomatitis (38.5%), retinal pigment epithelial detachment (RPED, 30.8%)	[89]
	Proof-of-Concept Study of AZD4547 in Patients With FGFR1 or FGFR2 Amplified Tumours	Phase II – Recruitment status unknown	FGFR2 (GC/GEJC) or FGFR1 (BC) gene amplification by FISH	GC/GEJC: RR was 33% (3/9) in FGFR2 amplified tumors BC: RR 12.5% (1/8) FGFR1 amplified tumors	Fatigue (71%), mucositis (41%), nausea (35%), and nail changes (24%). Asymptomatic RPED occurred in 1 pt	NCT01795768 [90]
	Efficacy and Safety of AZD4547 Versus Paclitaxel in Advanced GE or GEJC cancers (SHINE)	Phase II – Completed	FGFR2 status by FISH, and exploratory biomarker analysis. Marked intra-tumor heterogeneity	PFS for FGFR2 amplified arm: AZD4547 1.5M v. 2.3M for Paclitaxel	Stomatitis (20%), dry mouth (17.5%), RPED(15%)	NCT01457846 [143]
	Lung-MAP: Biomarker-Targeted Second-Line Therapy in Treating Patients With Recurrent Stage IV SqCLC (Arms I/III)	Phase II/III	FGFR1/2/3 mutation, fusion, amplification			NCT02154490
BGJ398 (Novartis)	BGJ398 for Patients With Tumors With FGFR Genetic Alterations	Phase II – Active, not recruiting	FGFR genetic alteration			NCT02160041
	A Dose Escalation Study in Adult Patients With Advanced Solid Malignancies	Phase I- Recruiting	FISH/CISH			NCT01004224
	SqCLC Expansion Cohort	Ib	FGFR1 amplification	SqCLC – PR in 2/17 within cutoff date and additional 2/17 afterwards, 3/17 SD	hyperphosphatemia, as well as stomatitis, alopecia, decreased appetite, and fatigue	[93]
	Urothelial Carcinoma	Ib	FGFR3 activating mutation	RR 9/25 (36%), within this was 1 CR (unconfirmed), and 8 PR (4 confirmed)	Hyperphosphatemia (42%), constipation (36%), fatigue (36%), elevated creatinine (36%)	[92, 144]
	BGJ398 in Non-Muscle-Invasive Urothelial Carcinoma of the Bladder	Pilot Study - Recruiting	FGFR3 activating mutation or gene fusion			NCT02657486
	A Phase 2 Study of BGJ398 in Patients With Recurrent GBM	Phase II - Recruiting	Amplification, translocation, or activating mutation in FGFR1/2/3/4			NCT01975701
	A Phase II, Single Arm Study of BGJ398 in Patients With Advanced Cholangiocarcinoma	Phase II – Active, not recruiting	FGFR2 gene fusion/ translocation	3/22 (PR), 15/22 (SD, 10 w/ tumor reduction)	hyperphosphatemia (50%), fatigue (42%), constipation (38%), cough (23%), and nausea (23%)	NCT02150967 [145]
	Study of the Efficacy of Single Agent BGJ398 in FGFR1-3 Translocated, Mutated, or Amplified Squamous Cell Carcinoma of the Head and Neck	Phase IIa – Not yet recruiting	FGFR1/2/3 mutation, amplification, or translocation) via DNA or RNA based assay			NCT02706691
JNJ 42756493 (Janssen)	A Study to Evaluate the Safety, Pharmacokinetics, and Pharmacodynamics of JNJ-42756493 in Adult Participants With Advanced or Refractory Solid Tumors or Lymphoma	Phase I – Active, not recruiting	FGFR2 or FGFR3 translocations	4/23 CR, 1/23 unconfirmed PR in glioblastoma, urothelial and endometrial cancer. 16/23 with SD	hyperphosphatemia (65%), asthenia (55%), dry mouth (45%), nail toxicity (35%), constipation (34%), decreased appetite (32%), and dysgeusia (31%).	NCT01703481 [97]
	An Efficacy and Safety Study of JNJ-42756493 in Participants With Urothelial Cancer	Phase II – Recruiting				NCT02365597 [146]
	Study to Evaluate the Safety, Pharmacokinetics, and Pharmacodynamics of JNJ-42756493 in Participants With Advanced Hepatocellular Carcinoma	Phase I - Recruiting				NCT02421185

RR = Response Rate; PR = Partial Response; CR = Complete Response ; SD = Stable Disease ; PFS = Progression free survival; GC = Gastric Cancer; GEJC = Gastroesophageal Junction Cancer; BC = Breast Cancer; SqCLC = Squamous cell lung cancer ; *Information up to date as of September 2016

## BIOMARKER DEVELOPMENT FOR PATIENTS WITH TUMORS HARBORING FGF/FGFR PATHWAY ABERRATIONS

As aforementioned, a recent study used next generation sequencing (NGS) to characterize frequencies of FGFR aberrations in nearly 5,000 solid tumor samples [23]. They found that 7.1% of malignancies demonstrated detectable abnormalities with the most common being gene amplification, followed by mutations, then rearrangements. FGFR1, as previously discussed, was most commonly affected. Within the cohort of malignancies analyzed, urothelial carcinoma exhibited the highest percentage of FGFR aberrancy (largely mutation, then amplification, followed by fusion) at 32%. Breast cancer, endometrial cancer, squamous lung cancers, and ovarian cancer followed in order of decreasing frequency [23]. Notably there was no evaluation of FGF ligand dependent signaling, highlighting that a subset of patients with FGF/FGFR pathway aberrations may still benefit from FGFR targeted therapy but were not characterized in this study. In their previous work, this group had noted FGF anomalies in approximately 14% of all malignancies [104]. Ultimately, when evaluating all FGF/FGFR aberrations (mutations, amplifications, rearrangements, etc.) they are detectable in nearly 20% of cancer histologies when assessed in combination [104].

Much of the pre-clinical and early clinical data come from trials in patient populations unselected for FGF/FGFR pathway abnormalities. The true response rates or clinical benefits for those whose cancers harbor FGF/FGFR abnormalities may be higher than observed in unselected patient populations. Many ongoing Phase I/II trials can be commended for aiming to select patients with specific FGF/FGFR alterations, and at this stage (appropriately so) there exist a variety in the methods including FISH, chromogenic *in situ* hybridization (CISH), quantitative real-time PCR, and NGS. Preliminary data, as discussed above, indicate tumor response with FGFR targeted therapies. However, detecting an aberration alone does not necessarily appear to predict tumor response. With FISH and IHC there is heterogeneity in expression of FGF/FGFR aberrations that may very well depend on the segment of tissue obtained at biopsy.

We must additionally consider that FGF/FGFR pathway alterations likely vary in their role depending on tumor histology and interactions with other oncogenic pathways. For example, one can learn from the development of EGFR inhibitors and the predictive biomarkers for response and resistance in both colon cancer and advanced squamous cell head and neck cancer. Recall that cetuximab was first approved for colorectal cancer with EGFR expression by IHC in 2004 [105]. It was later found out that EGFR expression by IHC in colorectal cancer did not correlate with response to therapy, and subsequent investigation led to the identification of the KRAS mutation conferring resistance [106]. Further study revealed that the best responders were also wild-type for both KRAS and NRAS [107]. This information is now included in the NCCN guidelines on the treatment of metastatic colorectal cancer prior to treatment with targeted EGFR therapy. Interestingly there is no specific methodology recommended (ie. sequencing or hybridization) in testing for these mutations [108]. In contrast, in advanced squamous cell head and neck cancers, testing for KRAS, NRAS, or EGFR expression did not correlate with a predicted response to EGFR targeted therapy. Cetuximab is currently approved in the metastatic setting for palliation as well as with concurrent radiation for definitive treatment of this malignancy without biomarker testing as a pre-requisite [109, 110]. This suggests that though a single pathway inhibitor works in two different histologies, the biomarkers for response or resistance may be different. The same may hold true for FGFR inhibitors.

As further studies unfold, we need to utilize multiplex molecular testing such as NGS to screen tumors harboring specific molecular aberrations of interest and increase the likelihood of detecting actionable FGF/FGFR alterations in each patient. Granted, even this technique may be limited by the location of biopsy and tumor heterogeneity especially in the setting of metastatic disease. While still a developing technology, a further biomarker analysis might include a measure of serum or urine circulating tumor DNA (ctDNA) in order to overcome this barrier. There are some studies that have identified a high concordance for actionable mutations between paired plasma and tumor specimens, especially for metastatic disease in non-small cell lung cancer, breast cancer, and colorectal cancer [111]. In the ongoing clinical trial NCT01795768 (a proof of concept Phase II Trial for AZD4547) as discussed above, the authors identified that all of the FGFR2 amplified gastric cancers that responded to therapy had elevated levels of circulating cell free plasma DNA [90]. There is a continued interest in urine ctDNA as well, as a completely non-invasive method of monitoring tumor dynamics in response to therapy. A recent study assessed the burden of ctDNA KRAS in a cohort of 13 patients with metastatic colorectal carcinoma (6 KRAS WT, 7 KRAS G12/13 mutants by tissue biopsy) using a commercial method for urine based ctDNA monitoring (PCR followed by NGS) which demonstrated stronger concordance with the clinical course and had fewer temporal fluctuations when compared to the dynamics of ctDNA KRAS in blood. In 1 patient, the urinary ctDNA increase even preceded radiographic disease progression by 2 months [112].

The accessibility of testing would likely allow for frequent monitoring of tumor evolution, and the presence of novel molecular alterations while actively receiving anti-cancer treatment may predict upcoming resistance to therapy. Already this has been described in a small cohort of patients with colorectal cancer initially demonstrating KRAS wild-type tumors, which subsequently were noted to have molecular alterations (*via* serum analysis) including KRAS, NRAS, EGFR, and BRAF after treatment with anti-EGFR therapies [113]. With a very sensitive testing approach you can pick up a resistant clone and theoretically modify your treatment regimen prior to disease progression. There still remain many questions with this technique, we first and foremost need to see that using ctDNA analysis to guide therapy leads to improved outcomes compared to molecular analysis from tumor biopsies. Whether this approach is relevant for FGFR targeted therapies remains to be seen with a validated translational study. Ideally, we could use this approach to monitor treatment response, disease recurrence, as well as pick up resistant clones in patients that have an FGFR alteration being treated with an FGFR inhibitor.

New trial designs and approaches are being developed in order to capture the many malignancies that may harbor an FGF/FGFR aberration. The Lung-MAP trial (umbrella trial) has been designed to study 4 targeted agents and 1 immunotherapy drug in patients with advanced stage IIIB/IV SqCLC with AZD4547 being one of the study drugs in target therapy group. It is a phase II/III, open label, multi-center study to assess the progression free survival and is still ongoing [114]. The inclusion is presence of FGFR 1/2/3 alterations, which are determined by FoundationOne test (NCT02154490). Phase II navigation clinical trials such as the BGJ398 basket trial for all solid tumors and hematologic malignancies and the NCI-MATCH are currently in development and will provide us with insight and direction as to better biomarker selection for response and resistance. The NCI-MATCH Trial (opened August 2015, NCT02465060) is designed as true histology agnostic basket trial for all solid tumors, with a plan to enroll rare cancers to account for at least 25% of the participants. The trial was paused several months after it opened due to rapid enrollment, and has since re-opened as of May 2016 with a plan to incorporate more sub arms (up to 24 total) which will include an FGFR inhibitor arm (AZD4547) [115].

## RESISTANCE MECHANISMS AND THE FGFR PATHWAY

Thus far we have discussed the use of FGFR inhibitors assuming that FGF/FGFR is the primary driver for oncogenesis in certain histologies or in certain molecular aberrations such as FGFR3 fusion in bladder cancer [116]. However, it is clear that the downstream signaling pathways in the FGF/FGFR cascade are highly interrelated, suggesting that FGF/FGFR inhibition should also be considered as a means to overcome acquired resistance to therapy in other malignancies. Additionally we must appreciate the evolving nature of cancer cells and the likelihood of resistance to FGFR inhibitors directly either by (i) compensatory signaling or (ii) *via* intrinsic gatekeeper mutations in the FGFR receptors themselves.

### FGF/FGFR INHIBITORS AS A MEANS TO OVERCOME ACQUIRED RESISTANCE TO VARIOUS CANCER TREATMENTS

Recently, acquired resistance to EGFR specific inhibitors in NSCLC mutant cell lines has been hypothesized to relate to the activation of the FGFR1-FGF2 autocrine loop [117]. FGFR is also involved in autocrine activation of STAT3 as a positive feedback in many previously treated cancer cells that are driven by oncogenes such as EGFR, ALK, MET, and KRAS [118].

In KRAS-mutant lung adenocarcinoma, in particular, strategies to inhibit the KRAS protein directly have not produced consistent results [119]. As such, the focus has shifted to targeting key downstream RAS pathway proteins including mitogen-activated protein kinase enzyme MEK (a component of the MAPK pathway), with one such drug being trametinib (MEK inhibitor). *In vitro* studies established that KRAS-mutant lung tumor cell lines treated with trametinib demonstrated an increase in FGFR1 receptor and/or ligand expression. This subsequently led to increased signaling through alternate pathways like AKT and ERK, which ultimately resulted in adaptive drug resistance [119]. Combining trametinib with ponatinib (multi-kinase inhibitor including pan-FGFR inhibition) resulted in a synergistic effect that allowed for continued inhibition of cell proliferation. This effect was appreciated in cell lines and xenograft mouse models of KRAS-mutant lung adenocarcinoma and KRAS-mutant pancreatic carcinoma, but was not as significant in KRAS wild-type lung cancer cells or KRAS mutant colon cancer. The effect was sustained with the use of AZD4547 and BGJ398 suggesting that it is inhibition of FGFR that likely accounts for the synergistic effect and not the inhibition of other RTK pathways. Interestingly, ponatinib alone had minimal effect on KRAS-mutant cells. The investigators made several conclusions, first that the compensatory response involving FGFR1 appears specific to particular KRAS-mutant cancer histologies. Secondly, they hypothesize that a combination of MEK and FGFR inhibition would likely be a valid approach in the treatment of KRAS-mutant lung cancer [119].

Much work has also been done investigating the relationship of FGF/FGFR with VEGF; preclinical models have shown that exposure to anti-VEGF treatment results in higher expression of FGF2 as the cancer progressed. Subsequent FGF blockade impaired further cancer growth [120]. This has been observed in both colorectal cancer and glioblastoma patients after exposure and failure to respond to anti-angiogenic/VEFG directed therapies [121]. In colorectal cancer cell lines demonstrating resistance to oxaliplatin or 5-fluorouracil (5-FU), a synergistic interaction between BGJ398 (silencing FGFR4) and these therapies was demonstrated to lead to reduction in cell growth and survival [122]. In breast cancer, FGFR1 amplification has been associated with endocrine resistance and poor prognosis [123]. These observations have led to the design of clinical trials evaluating malignancies that have failed standard therapies, with the idea that resistance *via* tyrosine kinase pathways may be a contributing factor in failure to respond. The interactions of the FGFR pathway and other known carcinogenic pathways suggest the role of FGFR signaling in acquired cancer therapy resistance by promoting cell survival and limiting overall drug response.

**Table 3:** Summary of Ongoing Clinical Trials Combining Selected Anti-FGFR Drugs and Existing Therapies

Malignancy	Study Title	Phase	Biomarker	Result/Response	Toxicity	Reference Number
**Breast Cancer**	Safety and Efficacy of AZD4547 in Combination With Fulvestrant vs. Fulvestrant Alone in ER+ Breast Cancer Patients (GLOW)	Phase I/II –Completed, enrollment suspended, concern for feasibility	FGFR1 polysomy (FISH4/5) or gene amplification (FISH6)	No participants completed trial		NCT01202591
	AZD4547 & Anastrozole or Letrozole (NSAIs) in ER+ Breast Cancer Patients Who Have Progressed on NSAIs (RADICAL)	Phase I/II - Recruiting	None			NCT01791985
**Solid Tumor**	Phase 1b Trial of BGJ398/BYL719 in Solid Tumors	Phase 1b – Active, not recruiting	Mutations to PIK3CA and alterations FGFR 1/2/3.	8/24 with PR (4 confirmed in urothelial, head&neck, melanoma, and anal cancer). 1 pt w/ FGFR3-TACC3 in urothelial cancer had complete shrinkage for 4M	Diarrhea (60%), fatigue (53%), nausea (48%), hyperphosphat-emia (37%), hyperglycemia (36%)	NCT01928459 [147]
**Lung Cancer**	Docetaxel With or Without FGFR Inhibitor AZD4547 in Treating Patients With Recurrent NSCLC	Phase I/II - Closed	FGFR1 gene amplification (score FISH6)		Lymphopenia (2/2), leukopenia (2/2), neutropenia (2/2) hypotension (2/2)	NCT01824901
**Urothelial Cancer**	Open-Label, Randomised, Multi-Drug, Biomarker-Directed, Phase 1b Study in Pts w/ Muscle Invasive Bladder Cancer (BISCAY) (MEDI4736+ AZD4547 v. AZD4547 alone)	Phase I – Not yet recruiting	FGFR3 - Mutation status of cancer associated genes in ctDNA			NCT02546661
**Gastrointestinal Stromal Tumor**	BGJ398 in Combination With Imatinib Mesylate in Patients With Untreated Advanced Gastrointestinal Stromal Tumor (GIST)	Phase I/II – Recruiting				NCT02257541
**Melanoma**	LGX818 and MEK162 in Combination With a Third Agent (BKM120, LEE011, BGJ398 or INC280) in Advanced BRAF Melanoma (LOGIC-2)	Phase II – Ongoing, Not Recruiting				NCT02159066
**Pancreatic Cancer**	Pan FGFR Kinase Inhibitor BGJ398 and Combination Chemotherapy in Treating Patients With Untreated Metastatic Pancreatic Cancer	Withdrawn prior to participant enrollment				NCT02575508

PR = Partial response ; NSCLC = Non-small cell lung cancer

*Information up to date as of September 2016

### PRIMARY RESISTANCE MECHANISMS TO FGF/FGFR PATHWAY INHIBITORS

In the development of a novel targeted therapy, we must also recognize the inevitability of acquiring resistance to the drug - either from up-regulation of compensatory pathways or innate mutations rendering the FGFR receptor resistant.

In a study using FGFR3-mutant cell lines, the investigators identified EGFR signaling as a key mechanism in limiting FGFR3 inhibition. In partially dependent FGFR3 cell lines, inhibiting FGFR3 resulted in a temporary down regulation of MAPK signaling that was bypassed by a prompt up regulation in EGFR signaling [124]. In EGFR dependent cell lines, they also identified that EGFR downstream signaling dominated, even in the presence of an activating FGFR3 mutation.

More recently, in SqCLC cell lines with FGFR1 amplification, investigators identified clonal cell populations that were resistant to treatment with AZD4547 or BAY1163877. They subsequently discovered the overexpression and activation of MET in these cell lines, and interestingly in cells treated with AZD4547 they identified MET gene amplification. In these AZD4547 treated cells, MET amplification was thought to lead to resistance through ErbB3 activation. The concurrent inhibition of MET signaling with the aforementioned FGFR inhibitors resulted in a reduction in cell growth. Additionally, when the investigators forced ectopic expression of MET in the SqCLC cells, they found that this conferred resistance to targeted FGFR inhibition. Overall, it appears that concurrent inhibition of MET and FGFR signaling pathways may provide synergistic benefit [125].

In discussing innate mutations, the “gatekeeper” mutation is responsible for the most common type of kinase inhibitor resistance, these are mutations of a residue located in the ATP binding pocket of the RTK. Learning from our prior experiences may allow for anticipatory guidance in the FGF/FGFR tale. Take for example the treatment of chronic myelogenous leukemia (CML). Since the approval of imatinib in 2001 targeting the constitutively active tyrosine kinase BCR-ABL1, we have seen the development of second and third generation tyrosine kinase inhibitors (TKIs) as increasing identification of resistance and intolerance unfolded [126]. A recent framework has been proposed to aid the clinician in selecting the appropriate TKI in their treatment of CML [127] . Imatinib, and both second-generation drugs, dasatinib and nilotinib, are currently FDA approved as first-line options for newly diagnosed CML in the chronic phase (CML-CP). For patients who fail this front line therapy, subsequent salvage therapy options include an alternative second-generation drug (the aforementioned, or bosutinib) or third generation ponatinib. These next generation TKIs are more potent and selective; thus far disease response patterns have been identified as relating to stage of disease, concurrent comorbidities, and BCR-ABL1 mutational status. Of critical importance has been the understanding that patients who develop the T315I “gatekeeper” mutation exhibit resistance to all the available TKIs except ponatinib.

A similar pattern of drug development and understanding has unfolded in the targeted treatment of EGFR mutant NSCLC or ALK-rearranged NSCLC. In treating EGFR mutant NSCLC, the first generation EGFR tyrosine kinase inhibitors gefinitib and erlotinib (approved in 2003 and 2004 respectively), followed by the second-generation afatinib, have been widely used in treatment of advanced disease [128]. However, disease progression frequently occurs after a median of 9 to 13 months of therapy, suggesting acquired resistance to treatment [128]. In fact, the most common acquired EGFR mutation leading to decreased survival has been found to be the “gatekeeper” mutation T790M [129], with nearly 50-60% of resistant cases demonstrating this anomaly [130]. Of course, there exist many other mechanisms for acquired resistance to therapy, including bypass signaling/compensatory activation of alternative RTKs, and downstream signaling molecules to name a few [131]. Continued development of EGFR inhibitor therapy led to the accelerated approval of the third generation EGFR inhibitor osimertinib for patients with metastatic EGFR T790M mutation-positive NSCLC that have progressed on or after previous EGFR inhibitor therapy [132]. Additional third generation agents continue to be in varying stages of clinical development.

In likewise fashion, there has also been significant progress in understanding and treating ALK-rearranged advanced NSCLC [133]. Nearly 5% of advanced NSCLC contain an ALK-rearrangement, for which crizotinib (multitargeted TKI of ALK, ROS1, and MET) was approved in 2010. As observed in the aforementioned stories, resistance to ALK blockade also emerges in time by multiple mechanisms including: ALK kinase mutations (30%) at L1196M (gatekeeper), F1174L, and G1202R, as well as activation of alternate oncogenes with resulting bypass signaling [133]. Second-generation ALK TKIs demonstrate more potent activity against ALK and ALK kinase mutants, and accelerated approval has allowed for ceritinib (2014) and alectinib (2015) to reach patients that have progressed on or are intolerant to crizotinib [134]. With ceritinib, there already exists some emerging data that ALK-G1202R and F1174V/C mutations confer some resistance to therapy [133]. As expected, ongoing study is evaluating newer second and third generation agents. Clearly, recognizing gatekeeper mutations is of clinical relevance in understanding resistance and realizing new actionable targets when designing and selecting the next line of therapy.

As highlighted in the above instances, an analogous pattern is being recognized in the family of FGFR receptors. Several pre-clinical studies have highlighted a significant gatekeeper mutation (FGFR1 V561M, FGFR2 V564I, FGFR3 V555M, FGFR4 V550M) that renders targeted therapy ineffective [135]. Preclinical cellular models harboring the FGFR3 V555M mutation have demonstrated resistance to AZD4547 [136]. Recently, the FGFR1 V561M gatekeeper mutation was characterized at a structural and kinetic level where a 38-fold increase in autophosphorylation of the receptor was demonstrated. Interestingly, the mutated receptor still maintained affinity for AZD4547 [137]. Subsequent generations of FGFR inhibitors will need to be able to circumvent these cellular defense mechanisms, and there exist two compounds FIIN-2 and FIIN-3 developed in preclinical studies that have demonstrated potency against wild type FGFR1-4 as well as receptors with gatekeeper mutations [135]. FIIN-3 in particular appears to have a more pronounced ability to inhibit both FGFR and EGFR signaling and was seen as more potent than FIIN-2 and BGJ398. This is of interest because aside from gatekeeper mutations, there is also evidence that FGFR inhibitor resistance can come from a switch to ERBB2/3 signaling (structurally related to EGFR) in models of FGFR3-dependent cancer cell lines [138]. When designing clinical trials, we should exclude tumors with the aforementioned gatekeeper mutations of FGFR known to confer possible resistance to currently available selective FGFR inhibitors.

## COMBINING FGF/FGFR PATHWAY INHIBITION WITH OTHER EXISTING CANCER THERAPIES

Based on the evidence, promising permutations for combined therapy may include FGFR inhibitors with endocrine therapy in breast cancer [123, 139], leading to trials such as AZD4547 & Anastrozole or Letrozole (NSAIs) in ER+ Breast Cancer Patients who have progressed on non-steroidal aromatase inhibitors. This is a Phase I/II trial that is actively recruiting (NCT01791985). It's likely that FGFR inhibitors would also be of clinical benefit in combination with EGFR targeted therapy, anti-VEGF therapy, and MET or MEK inhibitors given the known crosstalk among these oncogenic signaling pathways and the ability for cells to initiate compensatory signaling escape mechanisms when any one pathway is inhibited as previously discussed.

Interestingly, we know that many of the non-specific FGFR inhibitors such as lenvatinib and dovitinib are currently being used as treatment options in the clinical setting. This speaks to the notion that suppression of FGFR signaling in conjunction with other pathways is a valid approach; this may be part of the reason that singularly targeting the FGFR pathway has not resulted in meaningful outcomes warranting FDA approval at this stage.

## SAFETY AND TOXICITY OF FGF/FGFR PATHWAY INHIBITION

All the nonselective compounds currently being evaluated have shown toxicities related to VEGFR inhibition and deregulated angiogenesis, such as hypertension, cardiovascular events, and proteinuria. Moreover, the other most commonly reported adverse events include toxicities shared with other targeted agents, including gastrointestinal disorders (vomiting, diarrhea, decreased appetite) and skin reactions (stomatitis), and ocular effects (dry eye, retinal pigment epithelium detachment). Conversely, selective FGFR inhibitors show an “FGFR-specific” toxicity profile, including hyperphosphatemia thought to be related to FGF23 signaling [140]. At present, this has been managed with a low phosphate diet, phosphate binders, and diuretic therapy but there exist no strict guidelines on managing this side effect. A recent review suggested a framework for managing FGFR therapy hyperphosphatemia by first implementing dietary reduction in phosphate, followed by phosphate binders, and then manipulation in dosing schedule as needed. It was recommend that repeated episodes of phosphate ≥ 9mg/dL or simultaneous renal impairment would warrant discontinuation of therapy [140]. Preclinical models as aforementioned have proposed that increases in FGF23 and phosphate level may serve as markers for monitoring therapy, as an “on-target effect”. Ongoing clinical trials will likely continue to provide information regarding treatment monitoring, consider also the possibility of intensively checking calcium and magnesium. An additional point of interest is that a low phosphate diet would likely exclude foods such as chocolate, cheese, and ice cream, which realistically may not align with quality of life goals in patients presenting with advanced malignancies. A possibility exists that a preference not to comply with these dietary recommendations may preclude treatment with FGFR inhibitors altogether.

As is obvious in the field, the long-term consequences of continued FGFR suppression remain to be seen. Many have suggested that other drugs targeting downstream kinases, such as MAPK or PI3K/AKT, could offset intermittent FGFR inhibitor dosing. In the interim, methods for managing side effects will likely continue to emerge.

## CONCLUSIONS

The FGF/FGFR pathway demonstrates yet another mechanism that is critically involved in oncogenesis, thereby providing an actionable target for inhibition and exploitation of cell signaling. This comes with the promise to further the era of precision medicine. The traditional approach of tissue biopsy and FISH or IHC to identify mutation status may be inferior given the likelihood of tumor heterogeneity in advanced metastatic disease and also inherent technical limitations with this technique. Already in other known targetable mutations discussed above, such as EGFR in colorectal cancer or BCR-ABL1 in CML, the use of next generation sequencing (NGS) has proved invaluable in identifying not only actionable mutations, but also for screening “gatekeeper” mutations that may confer resistance to therapy. As selective FGFR inhibitors get closer to routine clinical use, we can learn from the past especially with regards to patient selection as it predicts response to therapy. As a whole, the advent of next generation sequencing with the concurrent use of ctDNA (blood or urine) have allowed us to better understand the biology of disease response and resistance in the development of FGF/FGFR inhibitors.

The Lung-MAP trial open through the NCTN (national clinical trials network) includes AZD4547 as one of the drugs in the target therapy group; it remains to be seen if FGFR amplification itself is a strong predictive marker of response to therapy in metastatic SqCLC. The investigators that designed the trial adopted NGS copy number variation to screen patients, however depending on the biomarkers, immunohistochemical assays may also be performed. The use of massive parallel DNA sequencing technology allows for noting differences in disease response based on FGFR mutation or fusion in lung, which may be important in a minority of lung cancers and this detail would be easily missed with FISH or IHC [114]. The NCI-MATCH trial (histology agnostic basket trial for all solid tumors) as aforementioned will also be including an FGFR inhibitor arm with AZD4547. This is an ideal platform to discover unusual responders to FGFR inhibitor therapy with the goal of identifying new and relevant FGF/FGFR alterations, especially with the addition of rare tumors that might otherwise not have enrolled in a clinical trial.

Despite the advances in drug design to include the second-generation selective FGFR inhibitors, the biology of FGF/FGFR signaling is complex and we have seen that response to therapy is dependent on a multitude of factors. At the present time, targeting FGFR fusion aberrations has demonstrated the best response; we see this in bladder cancer with encouraging results. Two phase I clinical trials (NCT01703481, NCT01004224) using the pan-FGFR inhibitors JNJ-42756493 and BGJ398 respectively, have reported partial response to therapy with FGFR3-TACC3 translocation or FGFR3 activating mutations primarily detected by FISH and IHC [141]. This is of interest given that the tumors in which FGFR3-TACC3 mutations have been identified (2.6% of urothelial carcinoma cases, 1.2-8.3% of GBM) are on the more aggressive end of the spectrum with overall limited treatment options [64].

Ultimately, we have the understanding that the FGF/FGFR aberrations do not behave uniformly across cancer histologies, suggesting both the ongoing need for understanding these differences as well as identifying the optimal strategy for detecting actionable mutations across a broad range of cancer types.
